# Comparison of differential metabolites in brain tissue of aged marmosets and serum of elderly patients after prolonged anesthesia

**DOI:** 10.3389/fnmol.2023.1134239

**Published:** 2023-03-24

**Authors:** Fengwei Zhang, Haoli Mao, Jiao Zhu, Ren Zhou, Lei Zhang, Hong Jiang

**Affiliations:** Department of Anesthesiology, Shanghai Ninth People’s Hospital, Shanghai Jiao Tong University School of Medicine, Shanghai, China

**Keywords:** metabolites, general anesthesia, aged marmosets, elderly patients, metabolomics, brain tissue, serum, oxidative stress

## Abstract

**Objective:**

To compare the differential metabolites in the brain tissue of aged marmosets after long-term anesthesia (≥ 6 h) and the serum of elderly patients by metabolomics methods.

**Methods:**

Six aged marmosets (≥ 8 years old) were divided into two groups: anesthesia and control. The aged monkeys in the anesthesia group were induced with 6–8% sevoflurane and 100% oxygen (2 l/min) for 1–2 min and maintained with 1.5–2.5% sevoflurane and 100% oxygen (2 l/min) for 6 h. In the control group (*n* = 3), anesthesia was only induced under the same conditions for 1–2 min. The prefrontal cortex tissues of the two groups of aged marmosets were collected for metabolomics detection. Twenty-nine elderly patients (≥ 65 years old) who had undergone surgical anesthesia for more than 6 h were enrolled. Serum samples were collected before and on the first day after surgery for metabolomics analysis. Differential metabolites were compared between human serum and marmoset brain tissue.

**Results:**

The changes in lactate and xanthurenic acid in the serum of elderly patients were consistent with those in the brain tissue of aged marmoset monkeys, that is, lactate was up-regulated and xanthurenic acid was down-regulated. However, serum levels of 5-methylterahydrofolic acid and leucine were down-regulated in elderly patients after anesthesia. In contrast, 5-methylterahydrofolic acid and leucine levels were up-regulated in the prefrontal cortex of aged marmosets compared with control marmosets. Furthermore, glycolysis/gluconeogenesis and pentose phosphate pathway were both significantly enriched in the prefrontal cortex of aged marmosets and serum of elderly patients after surgery.

**Conclusion:**

The changes of serum metabolites in elderly patients are not exactly the same as the metabolic changes of brain tissues in aged marmosets. The metabolic changes in serum lactate and xanthurenic acid levels can reflect brain tissue metabolism. The enrichment pathways of differential metabolites in the serum of elderly patients and the brain tissue of aged marmosets were partially the same.

## 1. Introduction

General anesthesia is the most widely used anesthesia method. Studies have revealed that general anesthetics can change the levels of metabolites throughout the body (Bannova et al., 2018). Our previous studies have revealed that anesthetics can change lipid metabolism in the brain tissue of aged marmoset monkeys (Mao et al., 2022). Different metabolites and metabolism-related signaling pathways are related to inflammation, cognitive function, and neurological diseases (Tripp et al., 2021). These changes may increase perioperative complications and mortality, affect postoperative recovery, and increase hospital stay in elderly patients. Therefore, the changes in metabolites in brain tissue caused by surgical anesthetics have attracted much attention.

Metabolomics research can improve our understanding of disease mechanisms, allowing us to prevent, delay, or improve disease onset and development (Tripp et al., 2021). However, due to objective constraints, we cannot directly obtain human brain tissue for metabolic analysis, and many experiments rely on obtaining human peripheral blood for indirect analysis. Furthermore, the blood–brain barrier (BBB) ensures the stability of the internal environment of the brain because it prevents many macromolecules from entering the brain (Abbott et al., 2010; Cai et al., 2018). Therefore, detecting peripheral blood metabolites may not accurately reflect brain tissue metabolism. In recent years, marmosets have been found to be more human-like than rodents in developmental processes and brain structure and function. Furthermore, the marmoset model has been widely used in neurological diseases, and some higher cognitive abilities of marmosets are comparable to those of macaques. In neuroscience, marmosets have attracted increasing attention as animal models (Okano et al., 2012). However, marmosets and humans are of different species. Therefore, evaluating whether the metabolite levels and changing trends in the brains of marmosets can reflect and represent the metabolite levels in human brain tissue and peripheral blood, and whether they are consistent with the changing trends in brain metabolites, has become the focus of research.

Hence, our study aimed to analyze the changes in human serum metabolites before and after surgery under general anesthesia and brain tissue metabolites in marmosets before and after prolonged anesthesia to explore the relationship between brain metabolic changes in non-human primates and human serum metabolites. Further, we analyzed whether human serum metabolites can represent brain tissue metabolism.

## 2. Methods

### 2.1. Participants

#### 2.1.1. Marmosets

All animal experiments were performed in accordance with the National Institutes of Health Guide for the Care and Use of Laboratory Animals.

The marmoset study was conducted in accordance with the guidelines and regulations of the Animal Care Committee of the Center for Brain Science and Intelligent Technology Excellence. The Animal Care and Use Committee of the Institute of Laboratory Animal Science approved this study (CEBSIT-2021035, Chinese Academy of Sciences). Laboratory Animal Use License SYXK [Shanghai] (2021–0003).

#### 2.1.2. Elderly patients

This study adheres to the principles of the Declaration of Helsinki. This prospective observational cohort study was conducted in the Ninth People’s Hospital, Shanghai Jiao Tong University School of Medicine, Shanghai, China, from July 2021 to October 2021. The Ethics committee of Shanghai Ninth People’s Hospital approved the study protocol (SH9H-2021-T120).

Inclusion criteria involved patients:

1. Undergoing neck and maxillofacial tumor resection under general anesthesia at Shanghai Ninth People’s Hospital, Shanghai Jiao Tong University School of Medicine,
2. With surgery duration longer than 6 h,
3. Aged 65 years and above,
4. Male or female, and
5. With American Society of Anesthesiologists physical status class I or II.

Exclusion criteria involved:

1. Preoperative history of mental illness and psychotropic drug use,
2. Alzheimer’s disease diagnosis,
3. Abnormal preoperative psychological scale assessment.

We also excluded (1) Postoperative Delirium (based on the 3D-CAM scale) and (2) emergency rescue history during the perioperative period to ensure the accuracy of the study. Participants signed written informed consent before the commencement of the study. The study was conducted in accordance with Strengthening the Reporting of Observational Studies in Epidemiology (STROBE) guidelines. The protocol was registered as a clinical trial under the registration number NCT05105451.

### 2.2. Methods

#### 2.2.1. Marmosets

Six marmosets over 8 years old were equally divided into sevoflurane anesthesia and control groups. The anesthesia group (n = 3, one female and two males) underwent 6-h anesthesia (induction with 6–8% sevoflurane and 100% oxygen (2 l/min) for 1–2 min and maintenance with 1.5–2.5% sevoflurane and 100% oxygen (2 l/min)). In the control group, aged marmosets (*n* = 3, one female and two males) were anesthetized with 6–8% sevoflurane and 100% oxygen (2 l/min) only for 1–2 min, and the prefrontal cortex tissues were collected for metabolomics analysis (Zhang et al., 2022).

#### 2.2.2. Anesthesia, surgery, and serum sample collection

All participants received standardized perioperative care. All patients underwent general anesthesia through tracheal intubation. Induction drugs included rocuronium (0.6 mg/kg), midazolam (2 mg), sufentanil (10–20 μg), and propofol (1–2 mg/kg). Antiemetic drugs such as durasetron were used as required before surgery. Airway gland secretion was inhibited with anticholinergic agents such as penehyclidine hydrochloride. Intravenous inhalation anesthesia was used during the operation, including propofol (2–6 mg/kg/h) combined with sevoflurane (1.5–2.5%) and remifentanil (0.05–1 μg/kg/min). Rocuronium and sufentanil were administered intermittently to deepen anesthesia and relax muscles as needed for surgery. The patient’s vital signs and bispectral index were monitored, and the depth of anesthesia was maintained accordingly. Patient-controlled analgesia with pentazocine was used for postoperative pain management.

In addition, 5 ml of blood was collected from participants before anesthesia (preoperative) and a day after surgery (postoperative). Samples were allowed to stand for 30 min at 20–24°C and then centrifuged at 3,000 rpm for 15 min at 4°C. Serum supernatants were collected and stored at −80°C.

### 2.3. Liquid chromatography–tandem mass spectrometry analysis

#### 2.3.1. Prefrontal cortex sample processing

External calibration against an internal standard was used for quantification. Standard prefrontal cortex homogenate was prepared by adding a diluted standard working solution to the marmoset prefrontal cortex homogenate. First, 20 μl of prefrontal cortex homogenate was mixed with a diluted standard working solution and 750 μl of methyltert-butyl ether. After standing for 30 min, 200 μl of pure water-MS level was added. After centrifugation at 15000 rpm for 15 min at 4°С, 700 μl of the supernatant was transferred into a new centrifuge tube. The supernatant was evaporated to dryness with a stream of nitrogen at 20°С–24°C. The dry residue was reconstituted with 100 μl dichloromethane and methanol (1:1 v/v) and transferred into the auto-injection bottle.

The sample extract was determined using reversed-phase chromatography with a mobile phase of water (containing 0.1% formic acid) and acetonitrile (containing 0.1% formic acid) at a constant flow rate of 0.20 ml/min. The injection volume was 1 μl, and the temperature of the autosampler was 40°С.

Multiple reaction monitoring mode was used for mass spectrometry detection. The heating electrospray ionization source parameters were as follows: 40 Arb sheath gas; 10 Arb of auxiliary gas; and the spray voltage was 3.5 kV (+)/2.5 kV (−). The temperature of the ion transfer tube was 320°C, and the evaporator temperature was 325°C. All mass spectrometer conditions were optimized for the quantitative detection of analytes.

#### 2.3.2. Processing of serum samples

We mixed 50 μl of serum and 300 μl of methanol with rotation at 4°C and 2,000 rpm for 10 min. After centrifugation at 12,000 rpm for 10 min at 4°C, 300 μl of the supernatant was transferred to a new centrifuge tube. The supernatant was concentrated to dryness using a stream of nitrogen. The dry residue was reconstructed with 50 μl of 2% acetonitrile. Subsequently, the reconstituted solution was mixed by spinning at 2,000 rpm for 10 min at 4°C, and centrifuged at 12,000 rpm for 10 min at 4°C. The supernatant was then transferred to an automated sampling vial.

### 2.4. Statistical analysis

Descriptive statistics were performed using methods applicable to each variable. Means and standard deviations were used for continuous variables with normal distributions. The median, 25^th^ percentile, and 75^th^ percentile were used for variables with non-normal distribution or ordered data. Differences between groups were tested using the Two-way ANOVA, Student’s *t*-test, Wilcoxon test, or Fisher’s exact test probability method, as appropriate. LSD method for multiple comparisons.

Metabolomics analysis included 58 serum samples and six prefrontal cortex samples. The metabolomics data included 182 serum metabolites and 194 brain metabolites. Online analytical tool MetaboAnalyst 5.0 was used for multivariate (multidimensional) statistical analysis. Multivariate statistical methods, such as principal component analysis (PCA), partial least squares discriminant analysis (PLS-DA), and orthogonal PLS-DA (OPLS-DA), were used to compare and analyze the whole metabolic spectrum and screen the differential metabolites. Multivariate statistical modeling was performed after data normalization. In addition, Two-way ANOVA, Student’s *t*-test and fold change were used to measure the significance of each metabolite. Differential metabolites were then selected using OPLS-DA variable importance of protection (VIP) > 1.0, Student’s test Raw *p*-value <0.05, and Fold Change >1.2 or < 0.83.

## 3. Results

The baseline characteristics of the six aged marmosets are presented in detail (Zhang et al., 2022). We enrolled 41 surgical patients during the study period. Patients with fewer than 6 h operation time and secondary or multiple operations after the first surgery were excluded. Finally, 29 patients were included in the metabolomics study. No significant difference was observed in age, sex, and American Society of Anesthesiologists classification. In routine laboratory, electrocardiogram, chest computed tomography, and other preoperative examinations, no significant difference was observed in the usage and dosage of rocuronium, propofol, sufentanil, sevoflurane, and other anesthetics used during the operation. In addition, vital signs were monitored during the procedure (Table 1).

**Table 1:** The basic characteristics of the elderly patients included in the experiment.

		Unit	Mean±SD (*n*=29)	Quantity and percentage
Baseline characteristics	Ages	(years)	72.41 ± 4.41	
	Male			19(65.5%)
	Female			10(34.5%)
	ASA II			29(100%)
	BMI	(kg/m^2^)	21.98 ± 2.72	
Diseases	Hypertension			11(37.9%)
	Diabetes			3(10.3%)
	Both			1(3.5%)
	Neither			14(48.3%)
Laboratory biochemical indexes	Hemoglobin	(g/L)	125.75 ± 18.21	
	Red blood cell	(*10^12^/L)	3.32 ± 0.53	
	White blood cell	(*10^9^ /L)	4.55 ± 0.99	
	Blood platelet	(*10^9^ /L)	207.52 ± 59.71	
	Total albumin	(g/L)	67.07 ± 5.24	
	Urea nitrogen	(mmol/L)	6.60 ± 2.43	
	Creatinine	(μmol/L)	65.64 ± 13.54	
	PT	(s)	11.10 ± 0.72	
	APTT	(s)	25.29 ± 2.25	
	Blood glucose	(mmol/L)	5.70 ± 1.73	
Intraoperative medication	Propofol	(mg)	1168.10 ± 566.21	
	Midazolam	(mg)	1.97 ± 0.18	
	Rocuronium	(mg)	60.86 ± 13.96	
	Sufentanil	(μg)	44.83 ± 8.66	
	Remifentanil	(mg)	2.18 ± 0.84	
	Sevoflurane	(%)	1.98 ± 0.09	
		(h)	7.35 ± 1.93	
Monitoring parameters	Anesthetic time	(min)	457.39 ± 82.12	
	Operative time	(min)	423.89 ± 80.72	
	Urine volume	(mL)	1737.90 ± 924.16	
	Blood loss	(mL)	517.24 ± 179.69	
Mean arterial pressure (MAP)	Preoperative	(mmHg)	100.30 ± 11.60	
	Anesthetic induction		88.20 ± 13.96	
	Intraoperative		75.84 ± 11.63	
	Postoperative		89.01 ± 10.53	
Heart Rate	Preoperative	(bmp/min)	72.38 ± 9.09	
	Anesthetic induction		71.48 ± 10.78	
	Intraoperative		60.76 ± 6.22	
	Postoperative		67.34 ± 9.49	
BIS	Preoperative		97.38 ± 1.73	
	Anesthetic induction		49.72 ± 9.79	
	Intraoperative		48.86 ± 4.87	
	Postoperative		53.55 ± 7.02	

The data are summarized as *n* (%) for categorical variables and mean ± SD for continuous variables. ASA, American Society of Anesthesiologists; BMI, body mass index; Both, Diabetes and hypertension; Neither, Diabetes nor hypertension were affected; PT, Prothrombin time; APTT, Activated partial thrombin time.

The PCA of the metabolite data of the prefrontal cortex of marmosets has been discussed previously (Zhang et al., 2022), and the top 15 differential metabolites after VIP analysis included xanthurenic acid (XA), 1-methyladenosine, and other substances (Supplementary Table 1).

**Figure 1:**
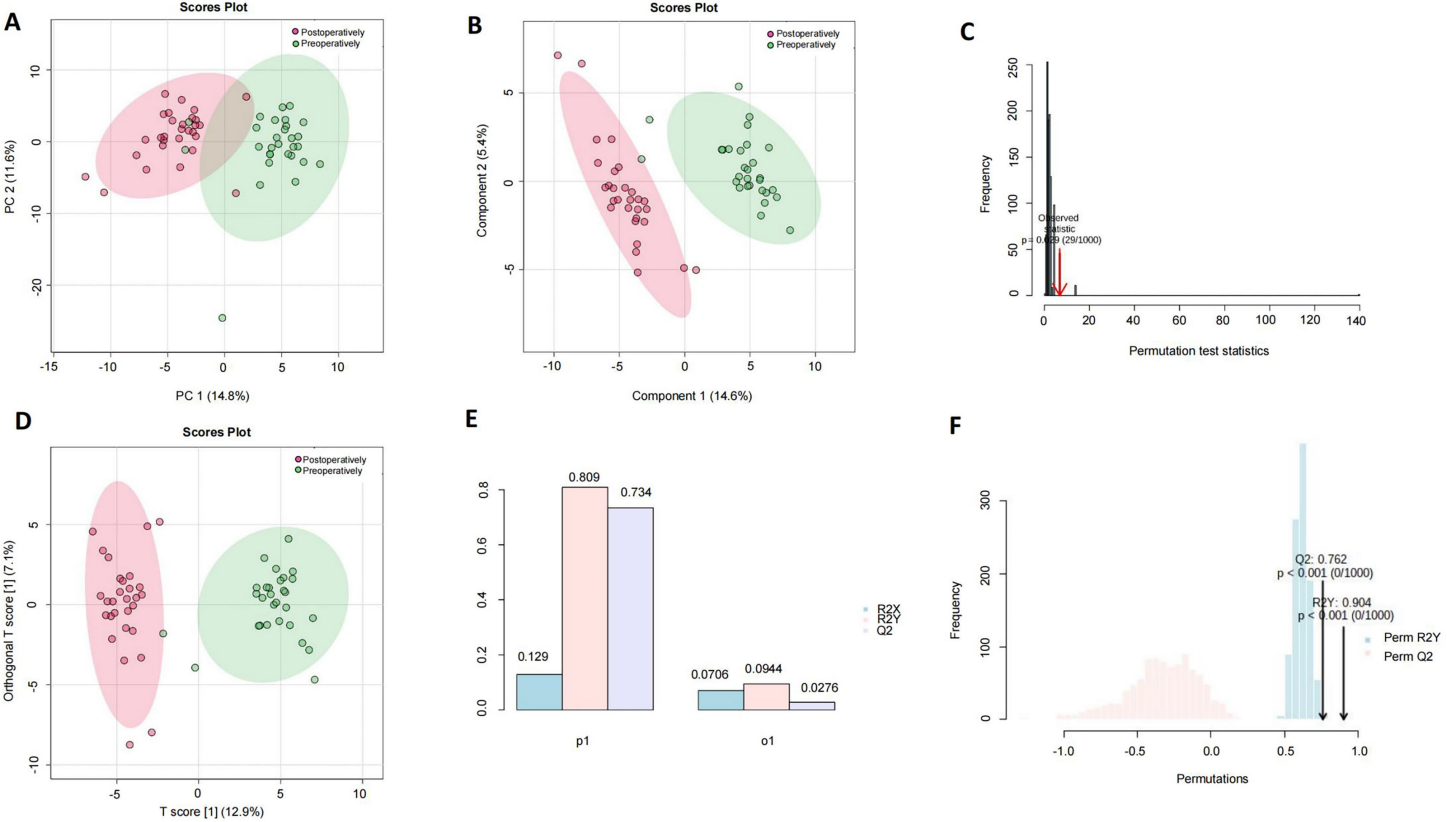
Analysis of differential metabolites in the serum of patients undergoing general anesthesia before and after surgery. The human preoperative serum is the control group (green dot), while the postoperative serum is the anesthesia group (red dot). **(A)** A plot of PCA scores for PC1 and PC2 extracted from human serum (*n* = 29). **(B)** Component differences between control and anesthesia groups in the OPLS-DA model of human serum metabolites. **(C)** PLS-DA model 1,000 permutation test results, the value of *p* was 0.029. **(D)** Control (green dots), and anesthesia (red dots) groups were completely separated on OPLS-DA two-dimensional score plot. **(E)** Human serum control and anesthesia groups OPLS-DA two-dimensional score plot model parameters. **(F)** OPLS-DA model 1,000 permutation test results, Raw *p*-value <0.01.

PCA score plots of PC1 and PC2 extracted from control (green dots) and anesthesia (red dots) groups were used for human serum (*n* = 29), and a clear trend of separation was observed between the groups on PCA two-dimensional score plots (Figure 1A). Compositional differences were observed between control (green dots) and anesthesia (red dots) groups based on the OPLS-DA model of human serum metabolites. A complete separation was observed on the PLS-DA two-dimensional score plot (Figure 1B), the PLS-DA model 1,000 permutation test results, and the Raw *p*-value was 0.029 (Figure 1C), indicating that the PLS-DA model may have overfitting. Control (green dots) and anesthesia (red dots) groups were completely separated on OPLS-DA two-dimensional score plot (Figure 1D), model parameters (Figure 1E), and OPLS-DA model 1,000 permutation test results with value of *p* <0.01 (Figure 1F).

**Figure 2:**
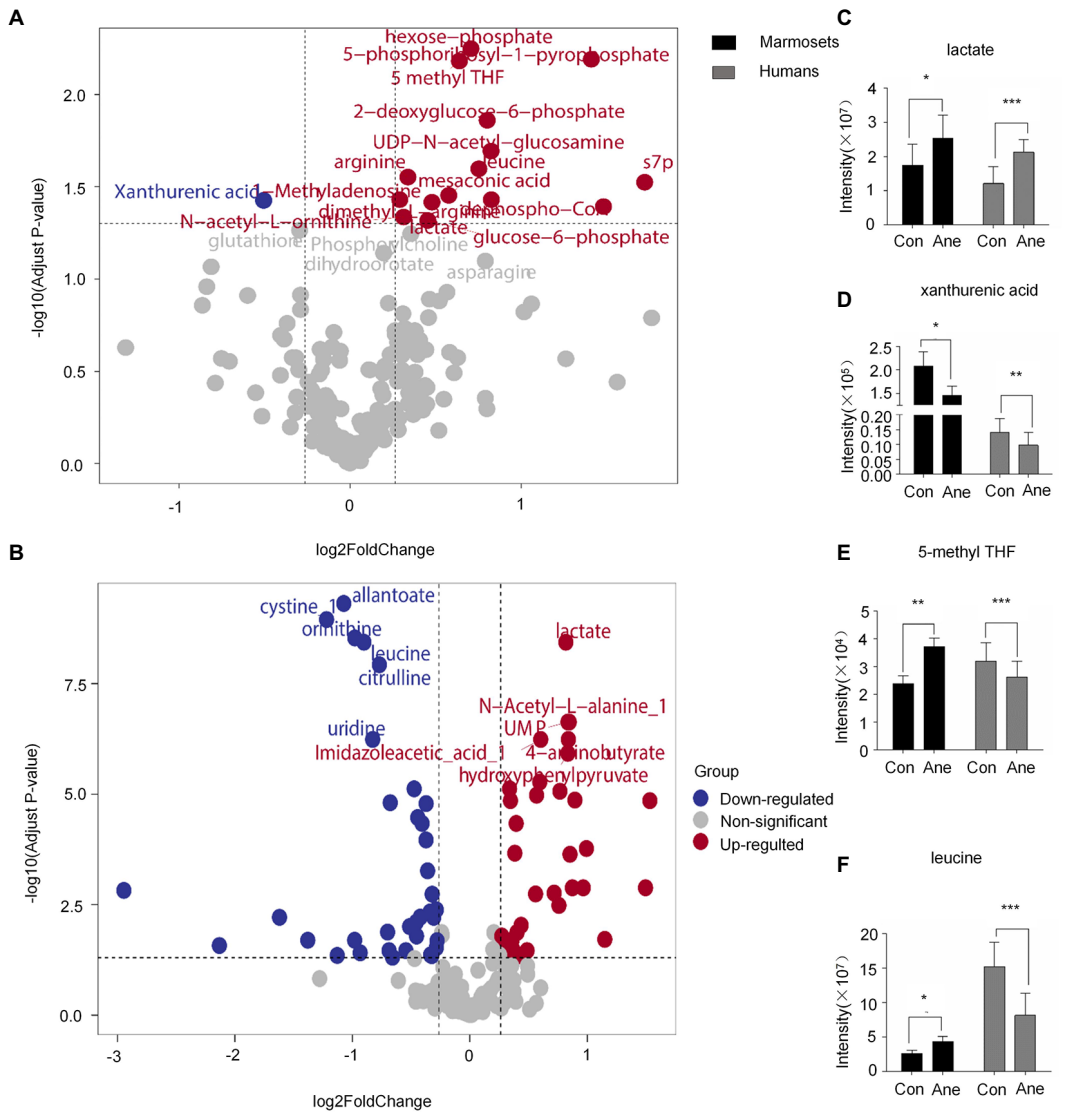
**(A)** and **(B)** Volcano plot of differential metabolite changes in marmoset monkey brain tissue and human serum after anesthesia, where the red dots indicate the metabolites that increased substantially in the anesthesia group compared with the control group (Fold Change >1.2, Raw *p*-value <0.05); Blue dots indicate metabolites that reduced significantly in the anesthesia group compared with the control group; Gray dots indicate metabolites that did not differ significantly in the anesthesia group compared with the control group (Fold Change <0.83, Raw *p*-value >0.05). **(C–E)**, and **(F)** Liquid chromatography–tandem mass spectrometry was used to detect the content of lactate, XA, 5-methyl THF, and leucine in the brain tissue of marmoset monkey and human serum. Sevoflurane anesthesia was defined as the anesthesia group, human preoperative serum as the control group, and postoperative serum as the anesthesia group. *p < 0.05 versus control; **p < 0.01 versus control; ***p < 0.001 versus control.

The differential metabolites VIP > 1 were analyzed using OPLS-DA (Supplementary Table 2). After statistical analysis, according to Raw *p*-value <0.05, fold change >1.2 or < 0.83, 16 of 194 differential metabolites in the prefrontal cortex of old marmosets were statistically significant. XA was down-regulated, and 1-Methyladenosine and N-acetyl-L-ornithine were up-regulated (Figure 2A). Of the 182 serum metabolites in the 29 patients who underwent surgery for more than 6 h, 60 were statistically different, with down-regulation of deoxycholic and cholic acids and up-regulation of alanine and phenylalanine (Figure 2B). Lactate, XA, 5-methylterahydrofolic acid (5-methyl THF), and leucine were expressed differently in the brain tissues of aged marmosets and human serum (Table 2). Further, Two-way ANOVA were used to investigate whether the changes of this four metabolites between marmoset and human similar (Supplementary Table 3). A interaction *p* < 0.05 means the changes between marmoset and human are different. The results of two-way ANOVA shown that only lactate show similar changes between two species (Interaction *p* = 0.7171) (Figure 2C). XA decreased after anesthesia in both marmoset brain and human serum. But in marmoset, the decline is even greater (Interaction *p* < 0.0001) (Figure 2D). For two other metabolites, the trend is quite different between marmoset and human (5-methyl THF Interaction *p* = 0.0005, leucine Interaction *p* = 0.0025) (Figures 2E, F).

**Figure 3:**
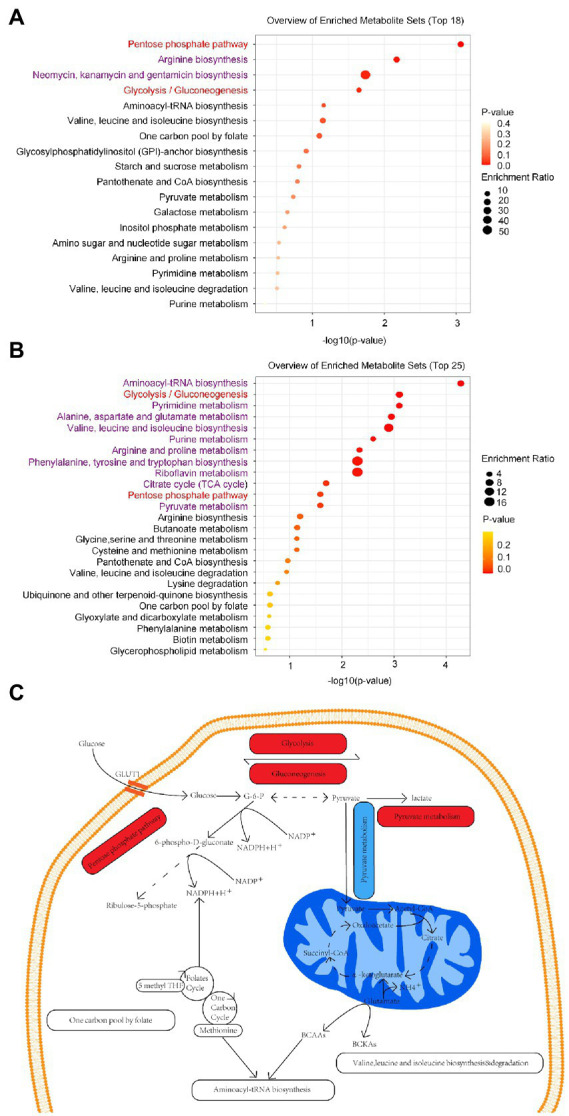
**(A)** There were 18 metabolic pathways enriched in the brain tissues of marmoset monkey, and the top 4 metabolic pathways were significantly enriched. **(B)** There were 25 metabolic pathways enriched in human serum, and the top 12 metabolic pathways were significantly enriched. The enriched pathways common to marmosets and humans are highlighted in red, and the remainder in purple. **(C)** Schematic diagram of the common enriched pathways in the neurons of human brain tissue. Activated pathways are shaded in red, inhibited pathways are shaded in blue, and other enriched pathways are shaded in white. G-6-P, Glucose-6-phosphate; BCAAs, Branched-chain amino acids: valine, leucine, and isoleucine; BCKAS, Branched-chain ketone acids; GLUT1, Glucose transporter 1.

In addition, we discovered that the marmoset differential metabolites were enriched in 18 pathways, including arginine, neomycin, kanamycin, and gentamicin biosynthesis (Raw *p*-value <0.05; Figure 3A). Human differential metabolites were enriched in 25 pathways, including purine and pyruvate metabolism (Raw *p*-value <0.05; Figure 3B). By comparison, humans and marmosets share eight metabolic pathways, aminoacyl-transfer ribonucleic acid biosynthesis, glycolysis/gluconeogenesis, valine, leucine and isoleucine biosynthesis and degradation, pentose phosphate pathway, one carbon pool by folate, and purine and pyruvate metabolism (Figures 3A,B). Two metabolic pathways were significantly enriched: glycolysis/gluconeogenesis and pentose phosphate pathway. In addition to purine metabolism, the metabolic pathways of seven common enriched pathways are shown in specific schematic diagram (Figure 3C).

## 4. Discussion

Our results suggest that the brain metabolism of aged marmosets is not identical to the serum metabolism of elderly patients in terms of the types, levels, and trends of differential metabolites or metabolic enrichment pathways after anesthesia. Lactate, XA, 5-methyl THF, and leucine can cross the human BBB; hence, changes in these four differential metabolites in the serum of elderly patients can represent the metabolic changes in their brain tissue. Only two of the four metabolites in the brain tissue of aged marmosets were consistent with changes in human brain tissue, while the other two were different. This indicates that brain tissue metabolism in aged marmosets are not identical to that of humans, and only part of them can represent the human brain tissue. The enrichment pathways of differential metabolites after anesthesia in aged marmosets and elderly patients were partially the same, demonstrating that metabolic pathways between marmosets and humans differed due to their differences in species. Therefore, we made inferences and conjectures regarding the differential metabolites and common enriched pathways observed in aged marmosets and elderly patients. Under physiological conditions, about 95% of dietary tryptophan is metabolized through the kynurenine pathway. The balance between kynurenic acid and XA is mainly due to kynurenine monooxygenase activity. Kynurenine monooxygenase inhibition reduced XA production and increased kynurenic acid concentration, which has neuroprotective properties (Schwarcz, 2016). The decrease in XA levels in human serum and brain tissue of aged marmosets after anesthesia could indicate the body’s self-protection of brain tissue against oxidative stress caused by surgical anesthesia.

**Table 2:** Fold Change, log_2_ Fold Change, Raw *p*-value and VIP value of four common differential metabolites in anesthetized human serum and marmoset monkey brain tissue.

Metabolites	Humans				Marmosets			
	Fold Change	log_2_Fold Change	Raw *p*-value	VIP	Fold Change	log_2_FoldChange	Raw *p*-value	VIP
Leucine	0.54	−0.90	<0.001	2.18	1.69	0.75	0.025	1.85
Xanthurenic acid	0.73	−0.46	0.002	1.34	0.70	−0.50	0.037	1.78
5-methyl THF	0.82	−0.28	<0.001	1.41	1.56	0.64	0.007	1.96
Lactate	1.76	0.82	<0.001	2.14	1.37	0.45	0.048	1.75

Factors such as surgery and anesthesia can lead to peripheral inflammation, increasing the permeability of the BBB. Changes in peripheral inflammation can also cause central anti-inflammatory responses, including lactic acid-induced hydroxyphenol receptor activation (Taylor et al., 2022). Increased endothelium-derived lactate levels after surgery and anesthesia can improve BBB integrity, thus, playing an anti-inflammatory role and providing energy for neurovascular peri cells. It also has neuroprotective effects (Lee et al., 2022). Pathological processes such as neuroinflammation (Luo et al., 2019), oxidative stress (Netto et al., 2018), and BBB destruction (Zhu et al., 2018) may be related to postoperative cognitive dysfunction. The same two metabolic enrichment pathways in aged marmosets and humans after anesthesia may be viewed as a self-protection mechanism of marmosets and humans in these pathological processes.

Prolonged fasting in surgical patients activates the gluconeogenic pathway, which uses glycogenic amino acids in the liver to produce glucose for the brain, an energy-intensive organ, and ketone bodies as an energy source. Valine is a glycogenic amino acid, leucine is a ketogenic amino acid, and isoleucine is a mixed type. As branched-chain amino acids, they play an important role in fat, amino acid, and glucose metabolism (Holeček, 2018). Furthermore, branched-chain amino acids are converted into end products (acetyl-coenzyme A and succinyl-coenzyme A) through a series of enzymatic reactions. It also participates in tricarboxylic acid cycle metabolism (Nie et al., 2018). Our results revealed that valine, leucine, and isoleucine are down-regulated in human postoperative serum, while leucine is up-regulated in marmoset monkey brain tissue, and branched-chain amino acids can cross the BBB (Neinast et al., 2019). One hypothesis is that leucine in the peripheral blood can cross the BBB and accumulate in brain tissue, suggesting that valine, leucine, and isoleucine biosynthesis and degradation may play an important role in fueling the brain during anesthesia. Another hypothesis is that the brain metabolism in marmosets may not represent that in humans. Our previous study revealed that the brain tissue of aged marmosets had increased lactate and an activated glycolytic pathway after prolonged anesthesia (Zhang et al., 2022). In this study, we observed an increased lactate in human postoperative serum, which reminded us that human brain tissue might undergo the same glycolytic activation process as marmosets during surgical anesthesia. In addition, the pentose phosphate pathway converts glucose-6-phosphate to pentose and produces aldehydo-D-ribose 5-phosphate and nicotinamide adenine dinucleotide phosphate (NADPH) (Tu et al., 2019). Our results indicate that 6-phospho-D-gluconate (Fold Change 0.85, log_2_FoldChange −0.23, Raw *p*-value 0.004, VIP 1.16) is down-regulated, which indirectly activates the body’s pentose phosphate pathway, a pathway that has long been studied as a branch of glycolysis. This pathway produces NADPH, which the body requires to fight oxygen-free radicals produced by drugs, food, or other stimuli. However, in recent years, the pentose phosphate pathway has also been related to neuroprotection (Tang, 2019), making us speculate that general anesthesia is an oxidative stress process that produces reactive oxygen species and causes nerve damage to human brain tissue. Two main sources of cellular oxidative stress in neuronal injury and disease are increased reactive oxygen species production caused by dysfunctional mitochondria (Nissanka and Moraes, 2018) and reactive oxygen species bursts caused by NADPH oxidases (Ma et al., 2018; Tarafdar and Pula, 2018). Therefore, general anesthesia is likely to cause mitochondrial dysfunction in human cells, which may explain why, even when the patient is provided sufficient oxygen during general anesthesia surgery, the pyruvate produced in the first stage of glycolysis is more likely to undergo lactate synthesis than shuttle into mitochondria for the tricarboxylic acid cycle to produce adenosine triphosphate. This hypothesis is supported by the upregulation of lactate levels in the brain tissues of anesthetized aged marmosets and postoperative serum of humans. An important active substance in folate metabolism is 5-methyl THF, which provides the active methyl group that drives the methionine cycle (Menezo et al., 2022). Some studies have suggested that folate metabolism could be a potential alternative source of NADPH (Chen et al., 2019). NADPH is produced *via* the oxidation of methylene-THF to 10-formyl-THF in mitochondria or the cytoplasm (Ducker et al., 2016). Therefore, we hypothesized that NADPH produced by one carbon pool *via* the folate and pentose phosphate pathway antagonized oxidative stress in brain tissue caused by anesthesia and surgery. Purine metabolism produces anti-inflammatory substances such as adenosine triphosphate and adenosine (Linden et al., 2019), aiding brain protection and stress reduction.

General anesthesia plays an important role in reducing the pain and psychological fear caused by surgery, but the unilateral factor of anesthesia is destruction rather than protection for the human body. Studies have revealed that repeated exposure of young rats to sevoflurane anesthesia for 2 h for three consecutive days induces a significant increase in the levels of interleukin-6, tumor necrosis factor, and other proteins in the brain, indicating that anesthesia induces an inflammatory response in young rats (Salaün et al., 2021). This is supported by the fact that the metabolic pathways activated in our aged marmosets after a single dose of anesthetic produce anti-inflammatory and anti-oxidative effects, as discussed previously. These metabolic pathways fully demonstrate the body’s ability to self-regulate and self-repair in the presence of external disturbances.

Most differential metabolites and enriched pathways between old marmosets and humans differ. Most differential metabolites in human serum do not appear in old marmoset monkey brain tissue, probably because:

- Humans are under the influence of both surgery and anesthesia, while marmosets receive only a single effect of anesthesia for the same period.
- Human anesthesia is a combination of intravenous and inhalant anesthesia, sedatives, analgesics, and muscle relaxants, while marmosets only receive sevoflurane inhalation anesthesia.
- Humans and marmosets have different tissues for metabolomics analysis. However, these differential metabolites could only be detected in peripheral blood but not in brain tissues due to their large molecular weight or the charge that could not penetrate the BBB.
- As there are species differences between humans and marmosets, the metabolic profile of marmosets is not completely representative of humans.

Our study had limitations. First, we could not set up the same number of marmosets for experimental controls as elderly patients due to their rarity. In addition, we did not analyze the specific activation or inhibition of the enriched pathways shared by elderly patients and marmosets but only indirectly inferred from the up-regulation or down-regulation of downstream substances, which can be further explored in future studies.

In conclusion, the metabolic changes in monkey brain tissue may partly represent the metabolic changes in human brain tissue. Metabolite levels in brain tissue and peripheral blood are not completely consistent. All metabolic pathways activated or inhibited by the body after surgery and anesthesia may represent self-regulation during external stress.

## Data availability statement

The original contributions presented in the study are included in the article/Supplementary material, further inquiries can be directed to the corresponding author.

## Ethics statement

The studies involving human participants were reviewed and approved by The Ethics committee of Shanghai Ninth People’s Hospital approved the study protocol (SH9H-2021-T120). The patients/participants provided their written informed consent to participate in this study. The animal study was reviewed and approved by The Animal Care and Use Committee of the Institute of Laboratory Animal Science approved this study (CEBSIT-2021035, Chinese Academy of Sciences).

## Author contributions

LZ and HJ contributed to the conception of the study and writing of the original draft. FZ, HM, JZ, RZ, and LZ contributed to the methodology of the study. LZ, HM, and HJ contributed to the writing, review, and editing of the manuscript. All authors contributed to manuscript revision, read, and approved the submitted version.

## Funding

National Natural Science Foundation of China (NSFC) Research grants (#82171173, #81970990, and #82071177). The Natural Science Foundation of Shanghai (grants 21dz1200205, 22ZR1437200, and 22YF1422500). Clinical Research Program of 9th People’s Hospital, Shanghai Jiao Tong University School of Medicine (JYLJ202221).

## Conflict of interest

The authors declare that the research was conducted in the absence of any commercial or financial relationships that could be construed as a potential conflict of interest.

## Publisher’s note

All claims expressed in this article are solely those of the authors and do not necessarily represent those of their affiliated organizations, or those of the publisher, the editors and the reviewers. Any product that may be evaluated in this article, or claim that may be made by its manufacturer, is not guaranteed or endorsed by the publisher.

## Abbreviations

XA: xanthurenic acid
5-methyl THF: 5-methylterahydrofolic acid
PCA: principal component analysis
PLS-DA: partial least squares discriminant analysis
OPLS-DA: orthogonal PLS-DA
BBB: blood–brain barrier
VIP: variable importance of protection
NADPH: nicotinamide adenine dinucleotide phosphate
