# Supplementary material for: Comparison of differential metabolites in brain tissue of aged marmosets and serum of elderly patients after prolonged anesthesia
Source: Front Mol Neurosci. 2023 Mar 24;16:1134239. doi: 10.3389/fnmol.2023.1134239 (PMC10081450; doi:10.3389/fnmol.2023.1134239)
Supplement: Supplementary file 1 [file Table_1.docx]

Supplement table 1 FoldChange, log_2_FoldChange, Raw *p*-value and VIP of brain tissue metabolites in Marmosets

|  | FoldChange | log_2_FoldChange | Raw *p*-value | VIP |
| --- | --- | --- | --- | --- |
| Xanthurenic acid | 0.7048 | -0.50471 | 0.037491 | 1.78355 |
| 1-Methyladenosine | 1.2231 | 0.29051 | 0.037145 | 1.760704 |
| N-acetyl-L-ornithine | 1.2424 | 0.31311 | 0.046134 | 1.732613 |
| arginine | 1.2642 | 0.33823 | 0.02805 | 1.819683 |
| lactate | 1.3707 | 0.45486 | 0.04818 | 1.75002 |
| dimethyl-L-arginine | 1.3936 | 0.47882 | 0.038374 | 1.816538 |
| mesaconic acid | 1.4914 | 0.57668 | 0.035243 | 1.785792 |
| 5 methyl THF | 1.5574 | 0.63913 | 0.006594 | 1.959039 |
| hexose-phosphate | 1.6317 | 0.70641 | 0.005651 | 1.980326 |
| leucine | 1.6854 | 0.75305 | 0.025301 | 1.850597 |
| 2-deoxyglucose-6-phosphate | 1.7433 | 0.80182 | 0.013811 | 1.951485 |
| UDP-N-acetyl-glucosamine | 1.7707 | 0.82434 | 0.020273 | 1.882733 |
| dephospho-CoA | 1.7727 | 0.82592 | 0.037142 | 1.752354 |
| 5-phosphoribosyl-1-pyrophosphate | 2.6582 | 1.4104 | 0.006449 | 1.980682 |
| glucose-6-phosphate | 2.7919 | 1.4812 | 0.040488 | 1.761259 |
| s7p | 3.2995 | 1.7222 | 0.029905 | 1.846366 |
