# Supplementary material for: Comparison of differential metabolites in brain tissue of aged marmosets and serum of elderly patients after prolonged anesthesia
Source: Front Mol Neurosci. 2023 Mar 24;16:1134239. doi: 10.3389/fnmol.2023.1134239 (PMC10081450; doi:10.3389/fnmol.2023.1134239)
Supplement: Supplementary file 2 [file Table_2.docx]

Supplement table 2 FoldChange, log_2_FoldChange, Raw *p*-value and VIP of human Serum Metabolites

|  | FoldChange | log_2_FoldChange | Raw *p*-value | VIP |
| --- | --- | --- | --- | --- |
| Deoxycholic_acid | 0.12993 | -2.9442 | 0.000283 | 1.366249 |
| Cholic_acid | 0.22814 | -2.132 | 0.009068 | 1.047656 |
| 2,3-dihydroxybenzoic_acid | 0.32518 | -1.6207 | 0.001447 | 1.32662 |
| shikimate | 0.38437 | -1.3794 | 0.006559 | 1.110151 |
| cystine_1 | 0.42943 | -1.2195 | 1.23E-11 | 2.258221 |
| allantoate | 0.47505 | -1.0738 | 2.67E-12 | 2.309877 |
| deoxyribose-phosphate | 0.50664 | -0.98098 | 0.006576 | 1.138923 |
| orinithine | 0.50743 | -0.97871 | 4.81E-11 | 2.182262 |
| 5-methoxytryptophan | 0.52385 | -0.93278 | 0.015099 | 1.089374 |
| leucine | 0.53541 | -0.90128 | 8.39E-11 | 2.176865 |
| uridine | 0.56322 | -0.82822 | 3.51E-08 | 2.028016 |
| citrulline | 0.58628 | -0.77034 | 3.88E-10 | 2.182183 |
| Glycerophosphocholine | 0.61536 | -0.70049 | 0.003415 | 1.120406 |
| 2-Aminooctanoic_acid | 0.62052 | -0.68844 | 0.013347 | 1.04874 |
| deoxyguanosine | 0.62507 | -0.67792 | 1.80E-06 | 1.754236 |
| sn-glycerol-3-phosphate | 0.6336 | -0.65836 | 0.020947 | 1.097605 |
| Methylterahydrofolic_acid | 0.68544 | -0.5449 | 0.012898 | 1.108483 |
| hydroxyproline | 0.70111 | -0.5123 | 0.002509 | 1.184239 |
| 2,3-diphosphoglycetic_acid | 0.71983 | -0.47427 | 6.24E-07 | 1.840023 |
| Xanthurenic_acid_2 | 0.72912 | -0.45577 | 0.001911 | 1.337204 |
| FAD | 0.73034 | -0.45335 | 0.004688 | 1.101713 |
| betaine | 0.735 | -0.44419 | 4.28E-06 | 1.599011 |
| CDP | 0.74641 | -0.42195 | 0.001367 | 1.178579 |
| indole_1 | 0.75457 | -0.40628 | 6.30E-06 | 1.59885 |
| tryptophan | 0.77207 | -0.3732 | 1.56E-05 | 1.568862 |
| Uric_acid | 0.77261 | -0.37219 | 1.98E-06 | 1.759335 |
| serine | 0.77932 | -0.35972 | 8.93E-05 | 1.49439 |
| isoleucine | 0.79105 | -0.33816 | 0.001015 | 1.31844 |
| Uracil | 0.79299 | -0.33463 | 0.017397 | 1.130643 |
| succinate | 0.79877 | -0.32415 | 0.018771 | 1.112923 |
| valine | 0.80075 | -0.32057 | 0.000374 | 1.420113 |
| D-glyceraldehdye-3-phosphate | 0.80813 | -0.30734 | 0.001422 | 1.202563 |
| lysine | 0.82012 | -0.28609 | 0.010638 | 1.089713 |
| 5-methylterahydrofolic_acid | 0.82079 | -0.28491 | 0.000887 | 1.414846 |
| proline | 0.8241 | -0.27912 | 0.006817 | 1.022261 |
| sarcosine | 1.2211 | 0.28817 | 0.005823 | 1.017792 |
| alanine | 1.2234 | 0.29086 | 0.005904 | 1.018908 |
| phenylalanine | 1.2641 | 0.33809 | 5.86E-07 | 1.678193 |
| purine | 1.2706 | 0.34548 | 1.50E-06 | 1.610796 |
| 3-methylphenylacetic_acid | 1.3033 | 0.38212 | 3.35E-05 | 1.599307 |
| dTDP | 1.3139 | 0.39389 | 6.19E-06 | 1.601368 |
| Phenyllactic_acid | 1.473 | 0.55878 | 0.000358 | 1.315099 |
| oxaloacetate | 1.4824 | 0.56791 | 9.86E-07 | 1.727662 |
| 2-aminoadipic_acid | 1.5099 | 0.5945 | 3.78E-07 | 1.764044 |
| Imidazoleacetic_acid_1 | 1.5188 | 0.6029 | 3.40E-08 | 1.857912 |
| CMP | 1.6437 | 0.71698 | 0.000333 | 1.195072 |
| S-adenosyl-L-homoCysteine | 1.7009 | 0.76626 | 7.55E-07 | 1.654147 |
| lactate | 1.7611 | 0.81648 | 1.00E-10 | 2.142366 |
| hydroxyphenylpyruvate | 1.7824 | 0.83383 | 7.82E-08 | 1.877411 |
| UMP | 1.7837 | 0.83487 | 1.00E-08 | 1.863065 |
| 4-aminobutyrate | 1.7888 | 0.83897 | 3.02E-08 | 1.785552 |
| N-Acetyl-L-alanine_1 | 1.7979 | 0.84635 | 1.02E-08 | 1.904819 |
| ADP | 1.8062 | 0.85297 | 3.65E-05 | 1.467637 |
| dATP | 1.83 | 0.87188 | 0.000232 | 1.324153 |
| glucono-D-lactone | 1.858 | 0.89373 | 1.34E-06 | 1.541064 |
| 3-S-methylthiopropionate | 1.9512 | 0.96439 | 0.000239 | 1.262825 |
| IDP | 1.99 | 0.99279 | 2.53E-05 | 1.510932 |
| riboflavin_1 | 2.2175 | 1.1489 | 0.005909 | 1.029995 |
| acadesine_1 | 2.8198 | 1.4956 | 0.000228 | 1.354335 |
| pyruvate | 2.8936 | 1.5329 | 1.53E-06 | 1.844598 |
