# Supplementary material for: Comparison of differential metabolites in brain tissue of aged marmosets and serum of elderly patients after prolonged anesthesia
Source: Front Mol Neurosci. 2023 Mar 24;16:1134239. doi: 10.3389/fnmol.2023.1134239 (PMC10081450; doi:10.3389/fnmol.2023.1134239)
Supplement: Supplementary file 3 [file Table_3.docx]

Supplement table 3 Two-way ANOVA analysis results of lactate, xanthurenic acid,5-methyl THF and leucine

|  | lactate | | xanthurenic acid | | 5-methyl THF | | leucine | |
| --- | --- | --- | --- | --- | --- | --- | --- | --- |
|  | F | P | F | P | F | P | F | P |
| species | 17.541 | <0.001 | 2538.003 | <0.001 | 0.281 | 0.598 | 34.492 | <0.001 |
| group | 20.402 | <0.001 | 98.810 | <0.001 | 2.118 | 0.151 | 3.588 | 0.063 |
| species*group | 0.169 | 0.682 | 77.164 | <0.001 | 13.406 | 0.001 | 9.966 | 0.002 |

F and P of the four metabolites were analyzed by two-way ANOVA.
